# Motivations regarding continuing or terminating pregnancy in women with high-risk pregnancies: a scoping review

**DOI:** 10.3389/fgwh.2025.1517669

**Published:** 2025-01-22

**Authors:** Mónica Antunes, Ana Galhanas, Ana Lúcia Vitorino, Sara Palma, Ana Frias

**Affiliations:** ^1^ Nursing Department, University of Évora, Évora, Portugal; ^2^ Nursing Research Platform Lisbon of Health Research Center (CIIS), Portuguese Catholic University, Lisbon, Portugal; ^3^ Department of Obstetrics, Évora Hospital of Central Alentejo, Évora, Portugal; ^4^ Department of Obstetrics, Garcia de Orta Hospital, ULS Almada/Seixal, Almada, Portugal; ^5^ Santarém School of Health Sciences, Santarém Polytechnic University, Santarém, Portugal; ^6^ Nursing Research, Innovation and Development Center of Lisbon (CIDNUR), Lisbon, Portugal; ^7^ Comprehensive Health Research Center (CHRC), University of Évora, Évora, Portugal

**Keywords:** decision making, high-risk pregnancy, motivation, pregnancy complications, pregnancy termination, review

## Abstract

**Background:**

The decisions of women with high-risk pregnancies to continue or terminate a pregnancy are complex and influenced by various factors. This scoping review synthesises the qualitative literature on the underlying motivations influencing these decisions.

**Aim:**

This analysis explores the underlying motivations that influence women's decisions regarding the continuation or termination of pregnancy, considering the challenges and dilemmas this population faces.

**Methods:**

This review was conducted following the Joanna Briggs Institute's methodology. No date restrictions were applied to the search. Titles and abstracts were screened to select original studies, and cross-checking was performed to avoid case overlap. We included studies that focused on the factors influencing women's decisions to either continue or terminate pregnancies when complications arose.

**Results:**

Eighteen studies involving women from different countries and cultural contexts were included. The review identifies four main themes driving these decisions: health considerations, religious convictions, social and political factors and ethical and moral dilemmas. Each theme interlinks to form a complex web of influences that significantly shape women's choices, illustrating how deeply personal, societal, and ethical contexts converge in these critical decisions. Significant emotional and cognitive factors, particularly hope, also play a crucial role. The findings highlight the complexity of the decision-making process and provide a deeper understanding of the personal, social, and spiritual dimensions involved.

**Conclusions:**

Multiple factors shape the complex decisions of women with high-risk pregnancies. Understanding these motivations is crucial to providing appropriate support and counselling. This review underscores the need for healthcare professionals to be aware of the diversity of factors involved and to adopt an individualized and context-sensitive approach in their practice, guiding their future actions.

## 1 Introduction

Pregnancy represents a pivotal period, often accompanied by significant physical and emotional changes. A naturally progressing pregnancy can escalate unexpectedly into a high-risk condition, marked by numerous potential complications (1). Good prenatal care becomes essential because pre-existing risk factors may arise and could compromise the maternal-fetal relationship. Early identification of complications that could jeopardize the quality of pregnancy enables healthcare professionals to plan interventions to prevent harm and ensure the healthiest possible outcome for the pregnancy (2).

The World Health Organization (WHO) recognizes high-risk pregnancy as a major public health challenge that demands priority attention. Nearly 22% of pregnant women encounter high-risk situations and driven by this concern and aligned with the Sustainable Development Goals (SDGs), countries have committed to a new target to accelerate the reduction of maternal mortality by 2030, emphasizing the urgent need for intervention (3).

A high-risk pregnancy may induce negative feelings due to the stress of an uncertain future, where expectations, adaptability, and past experiences significantly influence risk perception, which depends on various factors including knowledge of the situation, maternal attitude, degree of risk, psychological elements, and perspectives of healthcare professionals (4).

These women may develop feelings such as helplessness, fear, anger and anxiety, negatively experiencing behavioral, emotional, and affective aspects related to their family roles (5). Coping strategies for these women in the face of risk involve multiple challenges, where hope and resilience play essential roles in managing stress and mental health (3).

Pregnancy is not only unique due to physiological and psychological changes but also because of the significant responsibility it entails in making life-affecting decisions (6). Informed choice, a fundamental ethical principle in Western public health policies, plays a significant role in maternal health by enabling parents to make informed decisions about their care (7).

Decision-making in a high-risk context involves analyzing multiple options and probabilities, where factors related to survival and progress impact the decision-making process significantly (8). All women experiencing a high-risk pregnancy perceive risks based on their personal, family, and social interests (4), and studies on decision-making and informed choice suggest that decision-makers employ a rational process to decide between available options, influenced by uncertainty, risk, and utility (7).

Pregnancy can also lead to significant psychological shifts in social roles and physiological changes such as hormonal fluctuations, all of which can trigger cognitive and emotional changes in women, affecting their decision-making process (8). The decision to continue or terminate a pregnancy, whether voluntary or therapeutic, is complex and involves various factors in the decision-making process (9). An informed and conscious decision requires reflection on the advantages and disadvantages of different options and alignment with the couple's values (10).

Deciding on a reproductive option is crucial in a couple's life, yet little is known about the reasons and considerations that guide their decision-making process. Therefore, the objective of our study is to understand the underlying motivations that influence women's decisions to continue or terminate a pregnancy in a high-risk situation. We propose that delivering maternal health care rooted in values such as respect, understanding, and empathy is vital for deeper exploration into this subject. By fostering a more humanized approach to healthcare, we aim to enhance the quality of care provided to women and couples, cultivating an environment of trust, support, and relational care and ultimately improving nursing practice.

## 2 Materials and methods

The proposed scoping review was conducted following the methodology adapted from the Joanna Briggs Institute (JBI) guidelines for systematic reviews (11), which included defining the search strategy, selecting studies, assessing methodological quality, extracting data, synthesizing results, and evaluating confidence levels. To enhance clarity, the Population, Concept, Context (PCC) strategy was employed to frame the research question and guide the search strategy. Specifically, the “Population” involved women experiencing high-risk pregnancies, the “Concept” explored was the motivations and decision-making processes regarding the continuation or termination of these pregnancies, and the “Context” was high-risk health settings in hospitals and health centers.

Keywords derived from the PCC question such as “high-risk pregnancy,” “decision-making,” and “maternal health” were systematically used to ensure comprehensive database searching.

The protocol was registered prospectively with the Open Science Framework on 07 October 2024 [https://osf.io/g52us (accessed on 15 October 2022)].

### 2.1 Review question

The following question guided this scoping review: What are the underlying motivations influencing women's decisions to continue or terminate pregnancies in high-risk situations in the context of high-risk maternal-fetal health consultations in hospitals and health centers?

### 2.2 Inclusion criteria

#### 2.2.1 Participants

Women who have experienced high-risk pregnancies are defined as those with maternal, fetal or placental conditions that increase the risk of adverse outcomes for the mother and/or fetus during pregnancy, childbirth or postpartum. This review excludes any studies focusing on healthcare professionals.

#### 2.2.2 Concept

This review considers studies that explore the motivations and decision-making factors of women regarding the continuation or termination of pregnancy in high-risk situations.

#### 2.2.3 Context

High-risk maternal-fetal health consultations in hospitals and health centers are considered as the context to explore the motivations and decision-making factors influencing women's decisions to continue or terminate pregnancies in high-risk situations.

#### 2.2.4 Types of sources

This review included quantitative, qualitative, and mixed study designs. It also considered systematic reviews and meta-analyses of all kinds of study designs. However, the main focus was on qualitative studies due to the type of data we sought to explore more deeply.

### 2.3 Search strategy

The search strategy targets studies published in Portuguese, English, or Spanish, with no time limit. A three-step search strategy was implemented following the JBI's recommended approach for all reviews (11).

Initially, a preliminary search was conducted on the EBSCO platform, specifically in the PubMed and CINHAL databases, as well as on Google Scholar. This initial search was conducted to check for the existence of similar studies and to justify the need for this scoping review. Its findings confirmed that no comprehensive review exploring the motivations and decision-making processes of women in high-risk pregnancies currently exists, highlighting the importance of the proposed study.

This search utilized the keywords derived from the PCC (Population, Concept, Context) question to ensure a deeper understanding of the methodological process adopted. In the second step, a comprehensive search using all identified keywords and index terms was performed across all selected databases, employing Boolean operators like “OR” and “AND”. A detailed search strategy for PubMed has been developed based on these keywords and index terms (Supplementary Appendix A). In the third stage, the reference lists of selected full-text sources and those included in the review were examined for additional relevant studies.

The databases searched included CINAHL Complete, PubMed, Medline (via EBSCO), and Dynamic Health. Unpublished studies and grey literature were searched in Google Scholar and RCAAP (Portugal's Open Access Scientific Repository).

### 2.4 Study/source of evidence selection

Following the search, all identified citations were gathered and imported into Mendeley version 1.19.8 (Elsevier, Netherlands). The assembled bibliography was uploaded to Rayyan (Qatar Computing Research Institute, Doha, Qatar). After a preliminary test, at least two independent reviewers evaluated the titles and abstracts to assess compliance with the inclusion criteria. The second screening stage involved reviewing the references of selected studies to identify additional relevant studies. Studies were classified as included, excluded, or uncertain. Subsequently, full texts of “included” and “uncertain” studies were retrieved, and their full citation details were Imported into Rayyan (12).

**Figure 1:**
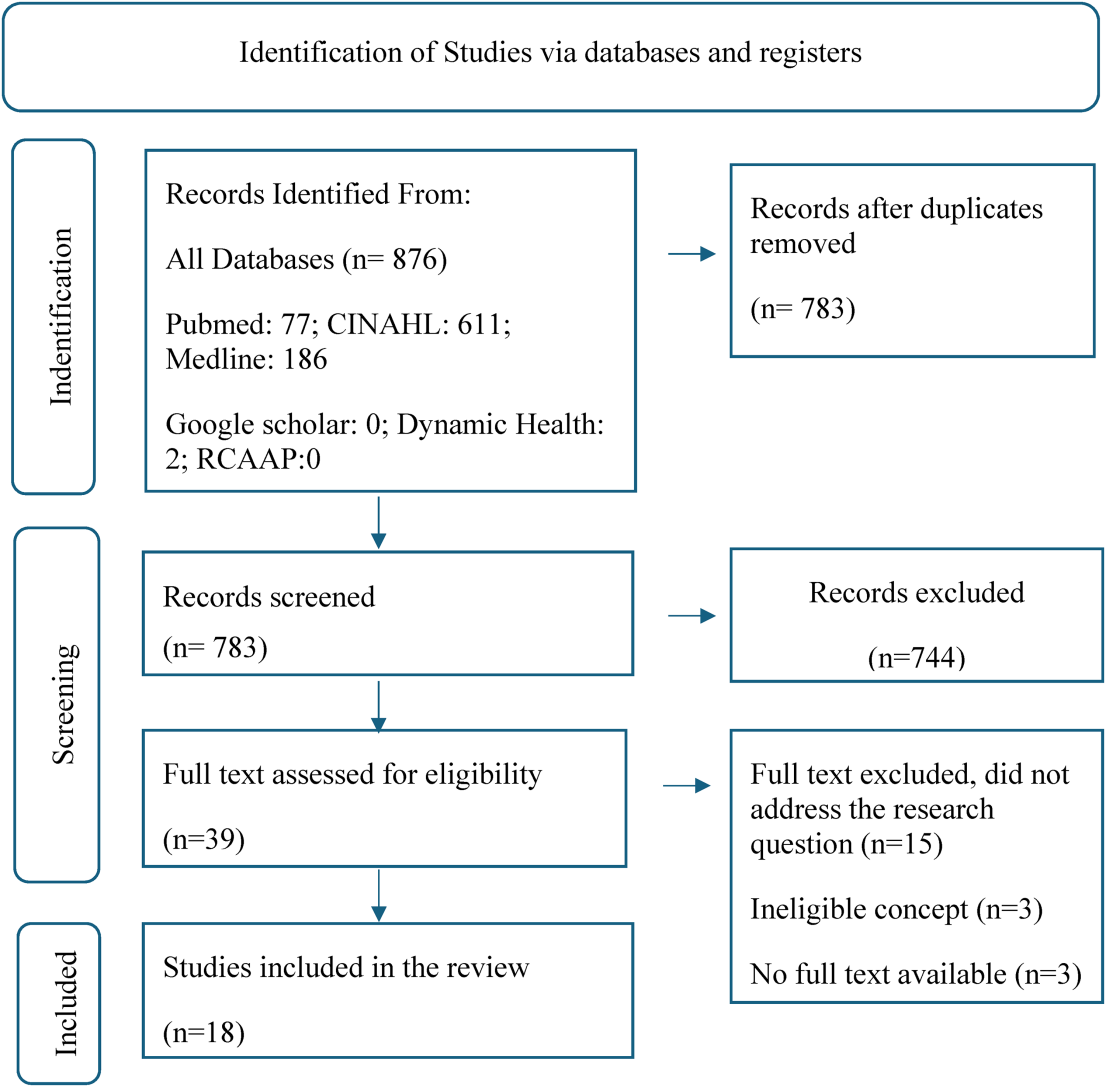
Flowchart of the selection and screening process of the systematic review articles according to the PRISMA method.

Two independent reviewers thoroughly assessed the full texts of selected citations against the inclusion criteria. The scoping review documented justifications for excluding studies that did not meet the inclusion criteria. Reviewers' disagreements during the selection process were resolved through discussion and a third reviewer. The flowchart of the selection and screening process of the systematic review followed the PRISMA-ScR method, which is the specific PRISMA for scoping reviews (13). The search results and study inclusion process were comprehensively reported in the final scoping review and presented in a PRISMA-ScR12 flowchart (Figure 1).

### 2.5 Data extraction

Following the review of titles and abstracts, duplicate entries and articles unrelated to the topic were excluded. Studies in Portuguese, English, and Spanish were considered, with no restrictions on publication date. The search process concluded on June 16, 2024, resulting in the exclusion of 783 articles based on relevance criteria. Additionally, 21 full-text articles were further eliminated—15 for not addressing the research question, three due to an unsuitable conceptual framework, and three owing to a lack of response from authors for full-text access—leaving 18 articles for detailed analysis (see Figure 1).

Data extraction was performed using calibrated forms that were tested by the team before use to ensure consistency and accuracy. Each article was independently reviewed by at least two reviewers, who recorded data on the study's population, context, concept, and results. Where necessary, data were recorded in duplicate to reduce bias and ensure reliability.

Inclusion and exclusion criteria were applied systematically, with decisions confirmed by consensus among the reviewers.

### 2.6 Analysis and presentation of results

The 18 studies eligible for SR (Scoping Review) are described in Supplementary Appendix B. The results are presented in narrative form. Considering the JBI guidelines, the synthesis of relevant data collected from each article was composed of the following elements: the identification of the article, motivations or experiences or/and expectations to continue or terminate the pregnancy, aims, study design, study population/sample, context, population characteristics, typology, and main results (13).

## 3 Results

The generated demand resulted in 876 titles. After applying the inclusion/exclusion criteria and excluding duplicate studies, 18 studies were eligible.

### 3.1 Characteristics of sources of evidence

The 18 articles were primary qualitative studies conducted across various countries. Additionally, two retrospective cohort studies and review studies are listed: one in Iran, three in the United States, five in Brazil, one in Ireland, one in South Africa, one in Portugal, one in Colombia, two in Poland, two in Denmark, and one in Turkey. The selected studies were published between 1997 and 2023. The women's ages ranged from 18 to 49. The sample sizes varied from 10 to one hundred seven women who used the services of specialized centers, public or private hospitals, and clinics for the specific medical condition presented.

Regarding the causes of the dilemma of terminating or continuing a pregnancy, it was found that only three studies referenced maternal conditions, such as being HIV-positive (14–16). The remaining articles portrayed indecision due to fetal causes, such as congenital fetal anomalies, with particular emphasis on Down syndrome (17, 18) and cases of anencephaly (17–20). According to MacCarthy, et al. for some women, being HIV-positive was the primary motivation to consider terminating the pregnancy (16). In contrast, for others, HIV was not a consideration in the decision to continue or terminate a pregnancy. Irani et al. note that, after detecting a fetal anomaly, women may be offered the option of terminating the pregnancy, which requires many informational and ethical considerations (18). According to several studies, the decision-making experience in a situation involving a fetus with a life-limiting condition is challenging for both men and women, causing significant emotional distress (14, 19, 21–23).

The perspectives of women who went through this high-risk pregnancy process contributed to the understanding of emotions surrounding loss (24). The decision to terminate a pregnancy for medical reasons is sensitive to religious and social determinants, especially in the current political situation where abortion may be prohibited; further, add that religious factors seem to be the key issue in decision-making (25).

When analysing the data, it was found that only three studies addressed the issue of hope in the context of a high-risk pregnancy (3, 18, 26). Irani (18) addresses the issue of hope as a category based on emotional and cognitive experience. Women who continued their pregnancies expressed feelings of hope but, at the same time, were concerned about the future. As stated by Kasnakoglu et al. and Antunes et al. (27, 28), pregnancy is a period of transformation, hope, and concern for women and their families. A positive pregnancy experience was an essential source of support and hope for women, where religion and spirituality were the most common attributes of hope found in the studies. For the families who decided to continue the pregnancy after an unfavorable diagnosis, this event had a positive meaning, providing an opportunity to experience motherhood in the prenatal context (29). The literature presents a perspective on healthcare professionals, in which the reframing of hope structure is proposed as a tool to support healthcare providers by enabling effective interventions grounded in women's experiences with this event (28).

### 3.2 Results of sources of evidence

According to the results of this review, women experiencing a high-risk pregnancy, whether due to maternal or fetal causes, face a dilemma between continuing or terminating the pregnancy. This dilemma requires significant reflective capacity from the couple. To answer the question: “*What are the underlying motivations influencing women's decisions to continue or terminate pregnancies in high-risk situations?”* we identified two main categories in our research, distinguishing the motivations of the woman/couple to continue and the motivations to terminate a high-risk pregnancy (Table 1).

**Table 1:** Categories distinguishing the motivations of the woman/couple to continue and the motivations to terminate a high-risk pregnancy.

Motivation to continue the pregnancy	Motivation to terminate the pregnancy
Being against abortion (20).	Risk to maternal mental health (17, 18).
Hope for a cure and treatment as a motivating factor and a source of strength to continue living (28).	Risk to maternal physical health (17, 25, 30).
Hoping for a miracle (20).	Fear and uncertainty due to evidence of fetal malformation (14, 15, 18).
Hoping the diagnosis will not be confirmed at birth (18).	Not wanting to bring a child with a disability into the world (19).
Motherhood shapes feminine identity and is perceived as more powerful than any challenge (28).	Financial conditions (16, 26, 31).
Religious reasons, specifically religions against abortion and the death of a new life (16, 21, 25, 27).	Possibility of illness or premature death of the mother (31).
Having a partner willing to assume paternal responsibilities and share the responsibility of raising the children (31).	Impact on the future of siblings (19, 25).
Correct counselling about the possibility of not transmitting HIV from mother to baby (16, 26).	Concern that the child will need special support throughout life or even institutionalization (19).
Family members stating it is too late to have an abortion (26).	Concern about bullying (19).
Public health burden for carrying out an abortion (26).	The idea of giving less attention to siblings (19).
Feeling unhappy about eliminating the product of one's own conception (26).	Concerns about the future due to increased domestic workload and struggles for social support (19).
Feeling sad about giving up the dream of starting a family (22).	Less freedom to pursue professional careers or carry out daily family activities (19).
Possibility and recourse to palliative care (14).	Belief that an HIV-positive mother will have HIV-positive children (26, 31).
Desire for a live birth and quality time with the baby (24, 29).	Fear of causing suffering to the baby (26).
Importance of creating memories (24, 29).	Fear of judgment, criticism, and lack of family support (25, 26).
Normal bodily experiences during pregnancy are seen as a ‘refuge’ for continuing the pregnancy despite a fetal diagnosis (29).	Influence of the partner's opinion, overriding the woman's own will (30).
Hope that the baby will be born alive (29).	Lack of social and financial support from the government for children and families with children with disabilities/health problems (25).
Importance of being a biological mother (29).	Fear of vertical transmission of HIV, either during pregnancy or childbirth (16).
Maximizing the well-being of the baby (29).	Family planning and the total number of children (16).
Recognizing the child as a person (29).	The type of congenital malformation (32).
The experience of pregnancy and childbirth brings feelings of real motherhood (29).	Number of living children (32).
Needing to experience this for personal life learning (20).	Fetal inviability (32).
Feelings of joy, love, hope, and fulfilment during pregnancy (29).	
Developing a relationship with their children before birth (29).	
The feeling of doing the best for their baby (29).	
Desire to embrace the potential burdens and blessings of raising a child with Down syndrome (19).	
Choosing the child, not the disease. Couples chose to embrace the child, not the illness, yet they also felt that the child was not something that could be ‘returned’ or ‘exchanged’ simply because they didn't match the envisioned future (22).	
Previous reproductive difficulties, making this their only chance at motherhood or parenthood in life (22).	
Acceptance and readiness for the uncertainties of life in general, including pregnancy, childhood, and family life (22).	
Lack of courage due to the meaning attributed to maternal love (30).	
Even in the face of fetal inviability, it is necessary for women to name and bury them with the family (30).	
Belief that the mother should not interfere with fetal life (30).	
Legislation on abortion and difficulties accessing it (16, 25).	

## 4 Discussion

This analysis delves into the deeply personal and complex factors that influence women's decisions to continue or end a high-risk pregnancy. This extended discussion aims to offer a broader and more critical examination of the socio-cultural and psychological dimensions that underpin these decisions. Drawing on insights from 18 studies conducted over the past 20 years across diverse countries and cultural settings, it sheds light on the emotional and practical challenges faced by women and their partners when making such a life-altering choice. The study identifies four main themes that significantly shape these decisions: health considerations, religious beliefs, social and political influences, and ethical and moral dilemmas.

### 4.1 Health considerations

The health of both mother and fetus emerges as a primary concern in the decision-making process. We compare these findings with existing literature on maternal health, highlighting that the complexity of healthcare decisions increases significantly under high-risk conditions. Women facing high-risk pregnancies often grapple with the potential risks to their mental and physical well-being, as well as the uncertainty surrounding fetal malformations (17, 25, 26, 30). The severity and nature of congenital anomalies play a crucial role in these decisions (14, 15, 18, 32). For women with HIV, the health considerations become particularly nuanced. While some view their HIV status as a key factor in considering termination, others find it has little impact on their decision to continue the pregnancy (16, 26, 31).

Interestingly, hope is a powerful emotional and cognitive factor in these decisions. Critically, the role of hope may vary significantly across different cultural contexts, affecting decision-making processes in diverse ways. As the author suggests (28), pregnancy is a transformative period filled with both hope and concern. This hope, whether for a cure or positive medical outcomes, can significantly motivate women to continue their pregnancies despite the risks (14).

### 4.2 Religious and spiritual factors

Religion often plays a central role in decision-making, providing moral guidance and strength to many women. An analysis of the impact of religious beliefs on healthcare decisions shows that these influences can have varying implications depending on cultural and geographic contexts. Religious beliefs, particularly those emphasising the sanctity of life, frequently motivate women to continue pregnancies even in adverse conditions (16, 21, 25, 27). The intertwining of hope with religious convictions is notable, with many women expressing hope for miraculous outcomes or viewing their pregnancy as a test of faith (18, 20, 28).

### 4.3 Social and political contexts

The impact of social factors, such as family dynamics, societal expectations, and the desire for motherhood, plays a substantial role in reproductive decision-making. This discussion reflects on how these social determinants interact with individual agency in high-stakes healthcare scenarios. The support of a partner and the emotional significance of biological motherhood often motivate women to continue their pregnancies (29, 31). However, financial limitations, fear of societal judgment, and the absence of support systems may lead some women to consider termination (16, 25, 26, 31).

In politically charged environments with restrictive abortion laws, these decisions become even more complex, as legal barriers and limited healthcare access create additional challenges (18, 25). These findings are consistent with other studies showing that socioeconomic factors and healthcare availability significantly influence maternal health outcomes and reproductive decisions (33). By reflecting on these complexities, we can better understand the interplay between policy and personal choice in maternal healthcare.

### 4.4 Ethical and moral dilemmas

The study reveals the profound emotional turmoil faced by women after receiving a fetal anomaly diagnosis. We reflect on the ethical implications of such diagnoses, suggesting that the decision-making process is heavily influenced by perceived societal norms and the availability of supportive healthcare services. Many struggle with concerns about the quality of life for a child with disabilities and the potential long-term impact on the family, including siblings (14, 19, 25). Yet, some couples embrace these challenges, framing their decision as “choosing the child, not the disease” (22). This demonstrates the deep emotional and ethical complexity of these choices.

Cultural and geographical differences add another layer of complexity to these ethical dilemmas. Women in different countries face varying societal norms and legal frameworks influencing their choices. The role of healthcare professionals in providing support and facilitating coping strategies becomes crucial in these contexts (18).

Cultural and geographical differences add another layer of complexity to these ethical dilemmas. By examining these global perspectives, we gain insight into how diverse societal norms and legal frameworks shape personal decisions. Women facing high-risk pregnancies require care that is both holistic and compassionate. Healthcare providers should be attentive to the wide range of factors influencing these decisions, including health, religious beliefs, social context, and ethical considerations. Recent research on shared decision-making in pregnancy emphasizes that building trust, offering thorough information, and respecting women's autonomy are essential in supporting them through these complex and deeply personal choices (23).

Ultimately, this discussion highlights the need for healthcare systems to consider a multifaceted approach to supporting women in high-risk pregnancy scenarios. This approach should integrate medical, ethical, social, and psychological support to address the complex realities faced by these women.

## 5 Conclusions and implications for practice

The decision of the woman/couple to continue or terminate a pregnancy after a medical diagnosis of high-risk pregnancy, whether due to maternal or fetal causes, is a difficult one to make, involving various factors such as personal characteristics, social, political, and even spiritual factors. All decisions require a profound moment of reflection, both individually and within the family, considering the weight of the consequences of that decision. Support from the multidisciplinary team is essential for a responsible attitude and a decision made with full awareness. Whatever the decision, it must always be respected by the healthcare team.

Our analysis points to several important directions for future research and clinical practice. First, there is a significant need to explore the role of hope-based interventions and their effectiveness in helping women navigate decision-making and develop coping strategies in high-risk pregnancies. Longitudinal studies examining the mental well-being of women who choose to continue vs. terminate these pregnancies are also crucial to understanding the lasting psychological impacts, including anticipatory grief in cases of severe fetal anomalies. Furthermore, investigating how family dynamics and cultural factors shape these decisions across different global contexts, particularly in countries with restrictive abortion laws and within indigenous communities, can offer valuable insights into the layered and complex nature of these experiences. The growing influence of digital media and online communities in shaping perceptions and decisions in high-risk pregnancies also merits thorough examination.

To enhance clinical practice, healthcare providers should develop comprehensive care models that address not only medical needs but also the emotional, psychological, and spiritual aspects of high-risk pregnancies. Improving cultural competence among healthcare professionals is crucial to effectively supporting women from diverse backgrounds. Additionally, educating women on critically evaluating information from media and online sources will empower them to make more informed decisions. By addressing these research gaps and implementing more holistic care approaches, healthcare providers can offer more tailored, empathetic, and practical support to women navigating the complex terrain of high-risk pregnancies.

## 6 Limitations

This review has several limitations. First, it focuses mainly on qualitative studies, which provide valuable, in-depth insights but limit the generalizability of findings to wider populations. Second, there is a notable gap in the literature, with only three studies examining the critical role of hope in high-risk pregnancies. Furthermore, the studies included span from 1997 to 2023, meaning that changes in medical practices and societal attitudes over time may affect the relevance of older findings in contemporary healthcare settings and decision-making processes. Additionally, cultural variations across the reviewed studies may influence reproductive decisions differently across regions, highlighting the importance of a culturally sensitive approach in interpreting and applying these findings globally.
