# Supplementary material for: Motivations regarding continuing or terminating pregnancy in women with high-risk pregnancies: a scoping review
Source: Front Glob Womens Health. 2025 Jan 22;6:1517669. doi: 10.3389/fgwh.2025.1517669 (PMC11794216; doi:10.3389/fgwh.2025.1517669)
Supplement: Supplementary file 1 [file Datasheet1.docx]

**Appendix A: Search strategy Search conducted on MEDLINE (Pubmed), search on 16/07/2024**

| *Search* | *Search Equation* | *Results* |
| --- | --- | --- |
| #1 | pregnant women OR maternal* OR expectant mother | 555,634 |
| #2 | Maternal decision making | 66 |
| #3 | high-risk pregnancy OR pregnancy complications OR obstetric complications OR medical condition OR obstetric health care (T +A) | 37,279 |
| #4 | Experiences OR motivations OR Incentives OR Reasons OR Driving forces OR Determinants OR Encouragements OR Inspirations OR Persuasions (T+A) | 480,950 |
| #5 | pregnancy termination OR pregnancy continuation (T +A) | 2,961 |
| #6 | 1 AND 2 AND 3 AND 4 AND 5 | 77 |
