# Supplementary material for: Motivations regarding continuing or terminating pregnancy in women with high-risk pregnancies: a scoping review
Source: Front Glob Womens Health. 2025 Jan 22;6:1517669. doi: 10.3389/fgwh.2025.1517669 (PMC11794216; doi:10.3389/fgwh.2025.1517669)
Supplement: Supplementary file 2 [file Datasheet2.docx]

| **Author(s) Year publication Country** | **Motivations or experiences or/and expectations** | **Aims** | **Study design** | **Study population/sample** | **Context** | **Population characteristics and typology** | **Main results** |
| --- | --- | --- | --- | --- | --- | --- | --- |
| “This is the child we were given”: A qualitative study of Danish parents’ experiences of a prenatal Down syndrome diagnosis and their decision to continue the pregnancy Stina Loua, Maja Retpen Lanthera , Natascha Hagenstjernea , Olav Bjørn Petersenb,c , Ida Vogel (2020). Denmark. | Prenatal screening for and diagnosis of Down syndrome (DS) and other chromosomal abnormalities has been increasingly routinized in many countries, which allows prospective parents to access information about their fetus and make reproductive choices. When DS is diagnosed, national termination rates are generally high. In Denmark, the termination rate is > 95% when prenatal screening leads to a DS diagnosis. Since the introduction of the national prenatal screening program in 2004, 23–35 children with DS have been born annually from approximately 60,000 live births. In most years, only 1–5 of these children were prenatally diagnosed, although this proportion has seemed to increase within the last few years. | To explore how parents of prenatally diagnosed children with DS experienced the diagnostic process and their decision to continue the pregnancy. | explorative research design with qualitative interviews | Parents | The participants were recruited from the National Association for Down Syndrome (NADS) in Denmark and from a Facebook group between December 2017 to February 2018. | Parents of a prenatally diagnosed child with DS and that the diagnosis had been made after the introduction of the national screening guidelines in 2004. | The couples reported several reasons and concerns that shaped their decision to continue the pregnancy.  - all couples recalled feelings of grief and sadness following the diagnosis and all described a process of mourning where they let go of the dream of a “normal” child and family future.  - Several couples were critical of the notion of “choosing to continue” and emphasized how they had not actively chosen or hoped for a child with DS.  - Several other couples emphasized that they had chosen the child, not the syndrome, but also that the child was not something that could be returned or exchanged because it did not fit their dream future.  - five couples had been through several years of reproductive difficulties prior to their pregnancy and some considered it a potential once-in-a-lifetime opportunity.  - the couples generally emphasized an acceptance and readiness for the uncertainties of life in general, including in pregnancy, childhood, and family life. Often, couples stressed that many unexpected things can happen in a child’s or a family’s life and that DS was not the worst that could happen.  - following the decision to continue the pregnancy, all couples experienced some concerns regarding their child’s future, such as cognitive development, potential additional diagnoses, and future life options. The couples worried about acceptance from their family and social networks, and were concerned about the attitudes and prejudices towards DS in society. All expressed some concern about the more distant future when they would no longer be around to care for their adult child with DS. During decision-making, some couples had been concerned that siblings would feel burdened by having a DS brother/sister; however, all couples—irrespective of parity—focused mainly on how a sibling with DS would add to their family in terms of teaching patience and empathy and for generating joy and happiness. Concerns about the parent’s own lives as adults (e.g., career) and as a couple (e.g., fear of divorce) were only mentioned in passing in some interviews and not at all in others |
| Concerns of Pregnant Women in “Prenatal Screening/Diagnosis” Practice and Termination of Pregnancy. Berna Tari Kasnakoglu, Mehmet Cakar, Zeynep Guldem Okem, Atakan Tanacan, Erdem Fadiloglu, Gokcen Orgul, Mehmet Sinan Beksac (2020). Ankara, Turkey. | the rate of acceptance of invasive prenatal diagnostic testing (IPDT) (amniocentesis, chorionic villus sampling, etc.) has decreased and the refusal of termination of pregnancy (TOP) has increased during recent years both in Turkey as well as in other countries. Recently increased political and social pressures in Turkey, such as highlighting the religious matters in everyday life by the government and the controversial attempts regarding the abortion law in 2012–13, have created substantial anxiety and stress in patients in their decision-making processes. | This study is designed to show the attitude, behaviour, and decision-making processes of patients in prenatal screening and diagnosis from a personal decision-making perspective. Our primary aim is to define the concerns and motivations leading to the refusal of IPDT and TOP procedures. We also aim to categorize these concerns and compare them with previous studies. | Qualitative study with questionnaires. | Pregnant women | prenatal screening at the Hacettepe University Hospital | 107 pregnant women referred for prenatal screening (combined test – first trimester screening tests for PAPP-A and HCG plus nuchal translucency, and a triple test for second trimester screening consisting of HCG, AFP and estriol) within the framework of the facility’s antenatal care programme between November 2017 and January 2018 | Undecided patients also had the highest level of concerns of all types, except for religious concerns. Religious factors seemed to be the key issue in decision-making.  Most people said NO, followed by those who were undecided. Only 10.3% of patients said they would undergo TOP if the IPDT result were positive. Again, undecided patients had the highest level of information on the procedures, whereas they had medium levels of concerns of almost all types. Those who were in favour of TOP had social, psychological, and support-related concerns, whereas those who were against TOP had high religious and trust-related concerns in addition to fears they had about IPDT (both from the procedure and the result of the test). |
| Moral dilemmas of women undergoing pregnancy termination for medical reasons in Poland. Kornelia Zaręba, Michał Ciebiera, Małgorzata Bińkowska & Grzegorz Jakie (2017). Poland. | Severe genetic and structural fetal defects are diagnosed in approximately 3% of all pregnancies worldwide. In Poland, that number amounts to 11,730 annually. Fifteen percent of all severe fetal defects end in a pregnancy termination. According to the Centre for Public Opinion Research Centrum Badania Opinii Społecznej survey of November 2011, 92% of respondents declared themselves to be ‘believers’ and 95% of the Polish population said they were Catholics, indicating the second highest, after Malta, number of Catholics per country in Europe. | Explored the religious views and dilemmas of Polish women making the decision to terminate a pregnancy. | Qualitative study with questionnaire. | Pregnant women |  | All patients deemed eligible for pregnancy termination in 2014 | The decision to abort a pregnancy for medical reasons is sensitive to religious and social determinants, especially in the current political situation in which abortion may become prohibited in Poland. The high response rate (100%) was probably the result of the patients’ attitudes: they repeatedly emphasised they were thankful for the help and empathy of the medical personnel and for being allowed to undergo the procedure. In Poland, the majority of centres use conscience clauses to justify their refusal to terminate a pregnancy. |
| On the path to interrupting the pregnancy or not: experiences of pregnant women with fetuses with anencephaly. Iulia Bicu Fernandes; Rozania Bicego Xavier; Paulo Alexandre de Souza São Bento; Andreza Rodrigues (2018). Brazil. | Anencephaly is a malformation characterized by the total or partial absence of the brain, the latter being the most common form; it is incompatible with extrauterine life, with the fetus only surviving for hours or days after birth. According to data from the World Health Organization, Brazil is fourth in the number of births of anencephalic fetuses (1/1600 live births). Of the pregnancies that are carried to term, approximately 75% of anencephalic babies are stillborn and the remainder die in the neonatal period. Facing the diagnosis of a child incompatible with extrauterine life leads parents to face great suffering and intense emotional experiences, in addition to health complications in the pregnant woman. | Understand the experiences of women with fetuses with anencephaly and identify the determining factors for the choice to terminate or not terminate the pregnancy. | Qualitative study and narrative method | Women | Public maternity hospital in the city of Rio de Janeiro. | Women over 18 years old and diagnosed with an anencephalic fetus, who underwent pregnancy termination or childbirth in a public maternity hospital in Rio de Janeiro. | Of the 12 women, only two did not want to interrupt and managed to keep their choices decided until the end. Their babies were born via cesarean section at term. For E5, the decision not to interrupt was closely related to what she reported as a lack of courage related to the meanings attributed to maternal love. In the case of E7, her desire was to give the baby a name and after it was born, that he could be buried with his family. Many women manage to find meaning in continuing with the pregnancy, even in the face of the unviability of the fetus and must be respected in their desire to remain pregnant. For some there is a need to give a name and bury them. Four women terminated their pregnancies for reasons related to their health (E2, E4, E10 and E12). In the case of E2 and E10, they did not delay in deciding to interrupt, due to the complications experienced. On the other hand, E12 decided to interrupt only in the third trimester, when complications began to compromise the couple's quality of life: I had direct arrhythmia and other complications – E10. Despite the presence of health complications, the decision to interrupt in the case of E4 proved to be complex, as she did not, in fact, want to interrupt the pregnancy, due to the belief that the mother should not interfere with the fetal lifespan |
| One Day You’re Pregnant and One Day You’re Not: Pregnancy Intewuption for Fetal Anomalies. Susan Hyde Bryar (1997). USA | Some authors do not include elective interruption of pregnancy for fetal anomalies with other types of perinatal loss, assuming that the voluntary nature of the decision separates these parents from those experiencing  miscarriage, stillbirth, or neonatal death (Menke &  McClead, 1990; Peppers & Knapp, 1980). However, the  acceptance of pregnancy interruption as a form of perinatal loss is increasing. | To systematically explore the processes operating within the experience of women undergoing 2nd-trimester pregnancy interruption for fetal anomalies. | Qualitative, descriptive, phenomenologic | Women | private outpatient perinatal practice associated with the medical center in the Northwest. | Women who had undergone pregnancy interruption for fetal anomalies in the 2nd trimester. | Making the decision is the negotiation of an acceptable decision about pregnancy interruption by examining one’s beliefs about abortion and quality of life and balancing those beliefs with the responsibilities and demands of the present. A decision to end a pregnancy involves consideration of many different factors. The reexamination of previously held beliefs, especially religious beliefs, the fulfillment of obligations to others, and the responsibility to the fetus are but a few of the influences affecting such a decision. Even after the decision is made, there is ongoing assessment and reassurance that pregnancy interruption was the right choice. Making the decision includes two subcategories: reexamining beliefs and fulfilling obligations. |
| Contemplating abortion: HIV-positive women’s decision to terminate pregnancySarah MacCarthya, Jennifer J. K. Rasanathanb, Ann Crawford-Robertsc, Ines Douradod and Sofia Gruskine (2013). Brazil. | Research on pregnancy termination largely assumes HIV status is the only reason why HIV-positive women contemplate abortion. As antiretroviral treatment (ART) becomes increasingly available and women are living longer, healthier lives, the time has come to consider the influence of other factors on HIV-positive women’s reproductive decision-making. | Exploring women’s pregnancy termination decision-making | Qualitative data were collected in semi-structured interviews | Pregnant women | State Reference Centre in Salvador, Brazil | HIV-positive women who knew their HIV status, currently or previously had had planned or unplanned pregnancies, and who were receiving HIV-related care at the only State Reference Centre (SRC) for HIV/AIDS in Salvador, Brazil. | Approximately half of respondents never considered abortion as an outcome for their pregnancies (n ¼ 13). While two women did not explain their rationale for not considering abortion, eight women stated that the pregnancy was desired, even if unplanned. Three objected to abortion on religious grounds. Of those women who did not consider terminating their pregnancies, three explained that they did not fear vertical transmission, especially after they gained knowledge about strategies to reduce the risk of HIV transmission. HIV-related concerns were otherwise not significantly related to these women’s wishes to continue their pregnancies.  O f the 12 women who did consider terminating their pregnancies, three women stated that their positive HIV status did not affect their desire to terminate their pregnancies. In contrast, five women contemplated this option solely on the basis of HIV-related concerns, particularly out of fear of vertical transmission during pregnancy or childbirth.  Four women considered abortion because of the aforementioned HIV-related concerns in addition to other factors, such as desired family size, economic status, partner influence provider influence and the lack of available, accessible, and acceptable abortion services. |
| Maternal risks and predictor factors for the termination of pregnancy in fetuses with severe congenital anomaly: experience from a single reference center in Brazil. Flavia Westphal, Edward Araujo Júnior, Suzete Maria Fustinoni & Anelise Riedel Abrahão (2016). Brazil.  ARTIGO 10 | the termination of pregnancy in fetal congenital anomalies is not allowed in several countries, mainly for religion reasons. In Brazil, only recently the termination of pregnancy in cases of anencephaly was allowed, although the termination of pregnancy for several other lethal malformations remains illegal. In these cases, when there is a diagnosis of fetal pathology without conditions of neonatal period survival and the couple wants the termination of pregnancy, only a judicial request allows the termination of pregnancy. | To describe the maternal complications in pregnant women with fetuses with several congenital anomaly as well as the predictor variables for the termination of pregnancy in a single reference center in Brazil. | retrospective cohort study | pregnant women | Fetal Medicine Discipline and deliveries in São Paulo Hospital. | All pregnant women with fetal infeasibility who performed their prenatal care in the Fetal Medicine Discipline and deliveries in the same institution (São Paulo Hospital). | From 94 pregnant women, 50 did not request the judicial authorization for the termination of pregnancy and six even with the judicial authorization, decided to maintain the pregnancy. The other 38 pregnant women, 25 requested judicial authorization and 13 already had legal reasons for the termination of pregnancy. The judicial authorization for the termination of pregnancy was obtained in 100% of the requested cases.  After simple logistic regression, the following variables influence the decision for terminating the pregnancy: type of congenital anomaly, the absence of maternal chronic disease, number of pregnancy, parity, living children, gestational age at diagnosis of fetal infeasibility and gestational age on arrival service. After multiple logistic regression, the following variables influenced the decision for the termination of pregnancy: type of congenital anomaly (OR: 18.59, CI95% 1.96; 175.87) and living children (OR: 0.45; 95%CI: 0.25; 0.80).  The type of congenital anomaly and living children were the most associated factors with the choice for the termination of pregnancy. |
| Emotional and Cognitive Experiences of Pregnant Women Following Prenatal Diagnosis of Fetal Anomalies: A Qualitative Study in Iran. Morvarid Irani; Talat Khadivzadeh; Seyyed Mohsen Asghari Nekah; Hosein Ebrahimipour; Fatemeh Tara (2018). Iran. | Nearly all (98%) pregnant women in Iran undergo an ultrasound screening at approximately the 18th week of gestation.2 The main purpose of this ultrasound screening is to calculate the gestational age, identify multiple pregnancies, and check for fetal anomalies. Pregnant women are often ill-prepared for the health of their unborn child in the case of abnormal findings, and experience several difficulties following the detection of fetal anomalies. | To explore the emotional and cognitive experiences of pregnant women following prenatal diagnosis of fetal anomalies in Mashhad, Iran. | Qualitative method and a conventional content analysis, with individual semi-structured in-depth interviews. | pregnant women | Two tertiary referral centers for fetal anomaly at Mashhad University Hospitals, Omolbanin Hospital and Imam Reza Hospital in Mashhad, Iran | Persian speaking parents with prenatal diagnosis of fetal anomalies at the gestational week of 12-27. All the pregnant women with a suspected or definitive diagnosis of fetal anomaly as per the ultrasound or the combined test (NT, free β-hCG and PAPP-A) were eligible for participation | Seven women (five primigravida and two multigravida) chose to terminate their pregnancy. These women were having a fetus with one of the following anomalies: Anencephaly, hydrops, trisomy 21 and diaphragmatic hernia. The remaining women continued their pregnancy. Four categories and 10 subcategories emerged from the data analysis about emotional and cognitive experiences of pregnant women following the prenatal diagnosis of fetal anomalies. The four categories themes included: Grief reactions during the time of diagnosis; Perinatal loss through a pregnancy termination; Fears of recurrence in future pregnancies; and a dilemma: hope and worries.  Pregnant women also expressed many concerns about continuing their pregnancy and the postnatal situation. For example, these worries included the status of continuing pregnancy, status of the baby after birth, threats of planned surgical treatment, and handling the care of other children during the period after birth. During the remainder of the pregnancy, when the couples searched to find more information about the detected defect, they could be re-traumatized due to the further bad news. |
| Malformação fetal com possibilidade de interrupção legal: dilemas maternos. Patrício SS, Gregório VRP, Pereira S, Costa R. (2019). Brasil.  ARTIGO 7 | Em 2016, nasceram 96.138 bebês em Santa Catarina, 836 foram a óbito no primeiro ano, sendo 203 por causa relacionada à “anomalia congênita”. Neste contexto, houve em 2012 a definição do Supremo Tribunal Federal Brasileiro (STF) quanto à antecipação terapêutica do parto em casos de anencefalia, descriminalizando-a. Fato historicamente discutido na sociedade e no campo da saúde. | Identificar os dilemas maternos sobre o diagnóstico de mal-formação fetal incompatível com a vida. | Método qualitativo do tipo exploratório-descritivo, com recurso a entrevistas semiestruturadas. | mulheres grávidas | O estudo ocorreu no Hospital Universitário da Universidade Federal de Santa Catarina (HUUFSC), a partir da entrada formal das mulheres no Ambulatório de Atenção à Saúde da Mulher ou entrada com diagnóstico confirmado de malformação fetal incompatível com a vida no Centro Obstétrico do HU. | Mulheres grávidas acima de 18 anos de idade, com diagnóstico confirmatório de malformação fetal incompatível com a vida, realizado através de ultrassonografia. Foram excluídas as mulheres grávidas com diagnóstico de malformação fetal incompatível com a vida que, a partir desta primeira consulta, não realizaram o seu seguimento no ambulatório do HU-UFSC | Das entrevistadas, cinco optaram pela interrupção legal, porém três estavam de acordo com a legislação vigente devido ao diagnóstico de anencefalia. Duas entraram com o pedido judicial, onde apenas uma teve parecer favorável para a realização.  As que escolheram a interrupção acreditaram que isto aliviaria tanto o sofrimento do feto como o seu. A decisão final em relação ao procedimento foi feita pelas mulheres, que contaram como apoio de seus parceiros. Contudo, foram momentos de muito desespero e sofrimento.  As três mulheres que optaram por continuar a gestação, também tiveram o apoio familiar e principalmente religioso, mantendo-se crentes em um milagre. Destas mulheres, uma era da religião espírita, uma evangélica e uma católica. Além disso, acreditavam que era necessário vivenciar essa experiência como um todo para uma aprendizagem pessoal de vida.  Obter a autorização judicial para interromper a gravidez foi difícil tanto emocional quanto burocraticamente para uma das duas participantes. A mulher se sentiu desrespeitada por ser obrigada a conseguir permissão legal num período tão doloroso, e ter que enfrentar julgamentos quanto ao seu direito de decidir sobre sua vida e sobre a gravidez. |
| Safety of the treatment for voluntary pregnancy termination by gestational age. Medellín, Colombia, 2013-2014. Bernal, Toro, Giraldo, Zuluaga & Osorio (2019). Columbia. | Maternal reasons for requesting pregnancy termination: The termination was requested due to risks to maternal health, including risks to mental and physical health, a history of sexual violence, and fetal malformations. Among the women with mental health risks, 10.3% had a psychiatric history, and two patients had a prior diagnosis of major depression. | To describe the safety of the medical-surgical treatment used in women seeking voluntary termination of pregnancy. | Historical cohort Study | Pregnant women | A referral institution in Medellín, Colombia. | Pregnant women at 16 weeks of gestation. Eighty-seven women were included, with a median age at the time of termination of 24 years (interquartile range [IQR] = 12). Of these, 69.0% were single, and 73.4% were unemployed. At the time of the procedure, the median age was 24 years. | All patients were affiliated with the Colombian General System of Social Health Security, with 48.3% under the state-subsidized regime, 49.4% under the contributory worker's regime, and 2.3% under other systems.  Maternal reasons for requesting pregnancy termination were as follows: 61.0% of terminations were requested due to risks to maternal health, with 55.3% of these due to mental health risks and 5.7% due to physical health risks. Additionally, 26.4% of requests were due to a history of sexual violence, and 12.6% were due to fetal malformations. |
| Hope Aspects of the Women’s Experience after Confirmation of  a High-Risk Pregnancy Condition: A Systematic  Scoping Review. Antunes Viana & Charepe. (2022). Portugal. | In the study of high-risk pregnancies with different medical problems, hopelessness  experiences are implicitly related to pregnancy worries, concerns about the child, future  pregnancy, relationships and support with others, and higher costs.  When the risk was associated with the diagnosis of HIV during pregnancy, the attribute  that emerged in all studies was fear of the cruelty of stigma, stereotyping, discrimination,  and judgment | Assess the state of knowledge regarding the  lived experience of hope among women facing high-risk pregnancies that may endanger  the health of the mother and/or fetus. | Review followed the Joanna Briggs Institute’s methodology. This review considered quantitative, qualitative, and mixed study designs for inclusion.  Systematic reviews and meta-analyses were also considered in this review. | Pregnant women | Women with high-risk pregnancies are usually referred to larger health centers for  better treatment. In this review, women were assisted in high-risk maternal–fetal health  consultations, which included the literature from any country or sociocultural setting. | Any patient with an age above 18, primigravida and multigravida, and in the second or third trimester of their pregnancy. This review excluded any studies focusing on healthcare professionals and pregnant women who were healthy and did not have any risk factors for high-risk pregnancies. | According to the results of the present scoping review, we found two main dimensions: women experiencing a high-risk pregnancy themselves and prenatal diagnosis. In both cases, the women  were in a dilemma between hope and hopelessness. |
| Demographic, Clinical, and Counseling Factors Associated with  the Selection of Pregnancy Termination Method in the Second  Trimester for Fetal and Pregnancy Anomalies. Maistrellis, Janiak, Hammel, Hurwitz, Delli-Bovi & Bartz. (2019).  United States of America | Reasons of fetal anomalies (e.g., structural and/or functional abnormalities of fetal chromosomes or cardiac, skeletal, neurologic, and other systems) and select pregnancy issues (e.g., intrauterine fetal demise [IUFD] and previable premature rupture of membranes | Despite women’s preference for induction of labor or dilation and evacuation for pregnancy termination in the setting of second trimester fetal or pregnancy abnormality, many women are not given a choice between delivery methods. | Retrospective cohort Study | Pregnant women | Such terminations are conducted in the United States using two methods, surgical dilation and evacuation (D&E) or medical induction of labor (IOL), both of which provide quite unique patient experiences of the pregnancy termination process. | Pregnancy termination at 17–24 weeks of gestation for fetal anomaly, intrauterine fetal demise, or premature previable rupture. | One hundred eleven women (21.6%) selected IOL and 403 (78.4%) selected D&E. Greater proportions of women of color (p < .01), lower education (p < .01), lower employment (p < .01), and lower status jobs (p < .01) selected IOL. Women selected D&E more often for chromosomal anomaly (p < .01). In adjusted analyses, women with intrauterine fetal demise (odds ratio [OR], 9.8; 95% confidence interval [CI], 2.8–34.7), premature previable rupture (OR, 110; 95% CI, 23.0–526.8), prior substance use disorder (OR, 35.5; 95% CI–2.7, 473.7), or counseling from obstetrics (OR, 3.3; 95% CI–1.3, 8.4), pediatrics (OR, 3.3; 95% CI–1.3, 8.6), or social services (OR, 12.6; 95% CI, 4.2–37.3) had higher odds of selecting IOL. |
| Post-diagnosis abortion in women living with HIV/Aids  in the south of Brazil. Pilecco, Teixeira, Vigo & Knauth.  (2015). Brasil. | The reproductive decisions of this group of women take into account not only the fact that they are HIV-positive and the possible risk of transmitting the virus to the fetus, but also individual factors, social aspects, as well as the cultural expectations placed upon them | To understand how the HIV diagnosis combines with other factors that influence the decision to abort. | Data  were collected during a crossover study of women | women | Public health services in Porto Alegre, Brazil. | Study of women aged between 18 and 49 years old. | The time between the diagnosis and abortion was 2 years or less for more than half of the women. For some, post-diagnosis abortion did not mean the end of reproductive life. The most  frequent reason for terminating pregnancy was to be living with HIV; however, only some of the women who stated having this motivation did not have post-diagnosis children. Changing partners between pregnancies was a recurring finding; however, in most pregnancies that ended in abortion, the women lived with their partners. |
| A qualitative exploration of HIV-positive pregnant  women's decision-making regarding abortion in Cape  Town, South Africa.  Orner, Bruyn, Harries & Cooper. (2012). South Africa. | Reasons to stop: Key issues to emerge were socio-economic hardship concomitant with concerns that pregnancy would undermine health, and fear of inflicting suffering on the baby and on other dependent children. Several women who were ‘ready to abort’ decided  against it after being counselled on prevention of perinatal HIV  transmission. | Understand HIV-positive pregnant women's decision-making regarding abortion in Cabo  City, South Africa | Qualitative study with interviews | Pregnant women | Study was conducted over a 13-month period in 2007/2008 at three public sector health facilities located in Cape Town. The facilities serve predominantly peri-urban working-class communities, and are broadly representative of the  types of HIV and abortion services in the area. | In-depth interviews were held with 24 HIVpositive women (15 had an abortion; 9 did not), recruited at public health facilities in Cape Town, South Africa. | Study results provide important insights, and any revision of reproductive health policy, services, counselling for abortion and HIV/AIDS care should address these issues. |
| Systematic Review of the Literature Parental Outcomes After Diagnosis of Fetal Anomaly.  Wool. (2011).  United States of America | Nonaggressive obstetric management, allowing natural birth without life-sustaining therapeutics, is an option for families. Families who experienced perinatal hospice/palliative care report positive  Feedback. | Review relevant articles with an aim to inform clinicians of parental experiences and outcomes after diagnosis of a fetal anomaly.. | A systematic review of the literature | Pregnant women |  | The review focused on patients given a diagnosis for fetal anomalies for the 40-year period from 1970 to 2010. | Results from this review of the literature emphasized that the experience of decision making for a fetus with a lifelimiting condition is a most difficult one. Both men and women suffered, and not always in the same way. |
| This pregnancy makes sense: Experiences of women who have decided to continue pregnancy after lethal fetal diagnosis Puzyna, Węgrzynowska, Ryś, Sys, Bączek & Baranowska. (2023)  Poland. | All women we interviewed stressed the personhood of their unborn babies. This was illuminated in the way women formed prenatal attachment to their babies and fought for and appreciated others recognizing  their babies’ personhood. Forming prenatal attachment All women developed a relationship with their unborn children. | Explore the experiences  and needs of women in Poland after lethal fetal  diagnosis who decided to continue with the pregnancy. | A qualitative approach based on semi-structured interviews | women | Women were recruited from the Warsaw Hospice for Children Foundation and the antenatal classes accredited by the Saint Sophia Specialist  Hospital in Warsaw. | Analyzed data collected from 10 women. The youngest participant was 27 years old and the oldest was 45 years old. The average age of all  the participants was 38.8 years and the median age was 38.5 years. All the women were college educated and lived in Warsaw metropolitan area. | For all of the participants, the continuation of pregnancy after lethal fetal diagnosis was an important and meaningful experience, allowing for prenatal motherhood. Prenatal motherhood was experienced on various levels, including physical, emotional, as well as relational experiences and a range of behaviors. The participants in our study referred to the moment when they found what they perceived as a supportive physician as a turning point that allowed them to focus on themselves and their babies instead of worrying about the practicalities of care. |
| Recasting Hope: A process of adaptation following fetal anomaly diagnosisq  Lalor, Begley & Galavan (2009) Irland. | For women who continue the pregnancy, evaluation of the psychological risks and benefits of knowing a fetal anomaly is present is essential, as routine screening means there is a real potential for large numbers to be affected. | Aiming to generate  theory to improve our understanding of the process of  adaptation following fetal anomaly diagnosis. | Analytical study. Theoretical framework of the process of adaptation following fetal anomaly diagnosis  based on women’s experiences of carrying a baby with a fetal abnormality up to and beyond the birth. | Pregnant women | The study was undertaken between October 2003 and September 2006. The fetal medicine unit of a major Dublin maternity hospital was chosen as the study site as it is also  a referral centre for many regional maternity units. | Thirty-one women continued the pregnancy and ten women travelled to the United Kingdom (UK) to access termination of pregnancy services not available within the state. | These forty-one women’s perspectives and experiences of this phenomenon have contributed to our understanding of the traumatic loss that accompanies this event. Recasting Hope is a theoretical rendering of the psychological disturbance and subsequent process of adaptation experienced by these women as they attempted to recover from this trauma. |
| Termination of pregnancy following a prenatal diagnosis of Down syndrome: A qualitative study of the decision-making process of pregnant couples. Lou, Carstensen, Bjørn, Palmhøj, Hvidman, Lanther &Vogel. (2018). Denmark | Knowledge of the couple’s initial decision may facilitate patient-centered communication during and after the diagnostic process. Couples may benefit from counseling to deal with grief and existential concerns. | Investigate the timing of the decision to terminate pregnancy following  a diagnosis of Down syndrome and the factors influencing this decision. | Semi-structured,  qualitative interview study. | Pregnant women | By participant choice, all interviews were performed in the homes  of the couples at a mean of 7 weeks after TOP. | 21 couples who had received a prenatal diagnosis of Down syndrome and decided to terminate the pregnancy. Participants were recruited from obstetric departments between February 2016 and July 2017. Data were analyzed using thematic analysis. | Five themes were identified: “initial decision-making”, “consolidating the decision”, “reasons and concerns shaping the termination of pregnancy decision”, “the right decision is also burdensome”, and “perceived influences in decision-making”. For most couples, the initial decision to terminate pregnancy was made before or during the diagnostic process, but it was re-addressed and consolidated following the actual diagnosis. Imagining a family future with a severely affected Down syndrome child was the main factor influencing the termination of pregnancy decision. The decision was articulated as “right” but also as existentially burdensome for some, due to fear of regret and concern about ending a potential life. The decision to terminate pregnancy was considered a private matter between the couple, but was refined through interactions with clinicians and social networks. |
